# LSD1 silencing contributes to enhanced efficacy of anti-CD47/PD-L1 immunotherapy in cervical cancer

**DOI:** 10.1038/s41419-021-03556-4

**Published:** 2021-03-17

**Authors:** Shengjie Xu, Xiaoyun Wang, Yongbing Yang, Yanli Li, Sufang Wu

**Affiliations:** Department of Obstetrics and Gynecology, Shanghai General Hospital, Shanghai Jiao Tong University, School of Medicine, Shanghai, China

**Keywords:** Tumour immunology, Cell signalling

## Abstract

Anti-CD47/PD-L1 immunotherapies aiming to enhance antitumor immunity are being intensively investigated and show promising results in cancer therapy; however, not all patients treated with these new drugs respond. Thus, developing new immunotherapy agents or combination treatments to enhance the efficacy of immunotherapy is an urgent challenge. Here, we found that LSD1 knockdown directly downregulated the expression of CD47 and PD-L1 through upregulating H3K4me2 levels in the *CD47* and *CD274* promoter regions. In addition, the LSD1/wild-type p53/miR-34a signaling axis was also involved in the regulation of CD47/PD-L1 expression by targeting the 3′ untranslated regions (3′UTRs) of *CD47*/*PD-L1*. Further, the results showed that an LSD1 inhibitor (ORY-1001) combined with anti-CD47/PD-L1 monoclonal antibodies inhibited tumor growth in an established subcutaneous xenograft model more effectively than a single blockade strategy. Collectively, these findings indicate that LSD1 inhibition enhances the therapeutic efficacy of PD-L1/CD47 blockade by reducing CD47 and PD-L1 expression in cervical cancer.

## Introduction

Cervical cancer is one of the three main malignant tumors of the female reproductive system worldwide, especially in China and other developing countries. Persistent high-risk human papillomavirus (HPV) infection is responsible for approximately 99.7% of all cervical cancers^1^. The major oncogenes E6 and E7 synergistically contribute to the progression of cervical cancer by inhibiting the activity of important tumor-suppressor proteins, including p53, retinoblastoma family members, and proteins containing PDZ domains^2^. Most patients with early disease (IA–IIA) are treated with surgery or radical radiotherapy. Locally advanced cervical cancer (IIB–IVA), which accounts for about two-thirds of all cases, has a 5-year survival rate of 40–50% despite appropriate multidisciplinary management.

Cancer immunotherapies, widely heralded as transformational for many cancers, are becoming viable options for selected categories of cancer patients. CD47, which is widely expressed on the surface of tumor cells, binds to SIRPα on the surface of macrophages to prevent them from phagocytizing tumor cells by creating a “do not eat me” signal^3^. Similarly, the programmed death ligand 1 (PD-L1; or CD274, B7-H1) is a critical “do not find me” signal to the adaptive immune system^4^. A certain correlation between these two seemingly independent signaling pathways has also been indicated. It was demonstrated that CD47 blockade therapy could activate the immune response of CD8^+^ T to tumors^5^, while PD-1 blockade therapy simultaneously promoted phagocytosis of tumor-associated macrophages^6,7^. CD47 and PD-L1 blockades are revolutionizing the treatment of various cancer types, but not all patients treated with these new drugs respond. Thus, developing new immunotherapy agents or combination treatments to enhance the efficacy of immunotherapy is an important challenge.

LSD1, the first histone demethylase to be discovered, is dysregulated in various tumors and is strongly correlated with the malignant progression of these tumors. Inhibition or knockdown of LSD1 has been demonstrated to suppress malignant biological behaviors of some solid tumors, including cervical cancer^2,8^. Inhibition of LSD1 has been proved to enhance the efficacy of tumor immunotherapy in melanoma and breast cancer^9,10^, thereby indicating a new direction for research on LSD1. However, the role of LSD1 in the immune microenvironment of cervical cancer is still unclear and requires further research. Immunotherapy is considered to be a new approach to the treatment of advanced cervical cancer, with some clinical trials showing encouraging results^11^. Meanwhile, in order to further enhance the efficacy of immunotherapy, combined treatment schemes have increasingly been investigated in clinical trials.

In our study, we validated the increase in expression of LSD1 during the progression of cervical cancer and confirmed the enhanced therapeutic effect against cervical cancer when combining CD47/PD-L1 blockade therapy with an LSD1 inhibitor. In addition, the LSD1-H3K4me2-CD47/PD-L1 and LSD1-wtp53-miR-34a-CD47/PD-L1 axes were found to explain the role of LSD1 in mediating cervical cancer immune evasion.

## Materials and methods

### Cell lines and cell culture

Human cervical cancer cell lines SiHa and C33A were initially obtained from the American Type Culture Collection (ATCC; Manassas, VA) and were maintained in our laboratory. Cells were cultured in 1:1 Dulbecco’s modified Eagle’s medium/F12 (Gibco, Auckland, New Zealand) with 5% penicillin–streptomycin and 10% fetal bovine serum (FBS; Gibco, Gaithersburg, MD, USA). In addition, TC-1 cells transformed with HPV16 E6 and E7 were purchased from Shanghai Institutes for Cell Resource Centre, Chinese Academy of Sciences and cultured in complete RPMI 1640 (Gibco, Auckland, New Zealand) with penicillin–streptomycin and 10% FBS (Gibco, Gaithersburg, MD, USA). All of the above cell types were cultured at 37 °C in a cell incubator with 5% CO_2_.

### RNA interference (RNAi) and gene transfection

The LSD1 small hairpin RNA (shRNA) plasmids were designed and synthesized by Genomeditech (Shanghai, China). The target sequences were as follows: 1# CCGGGCACCTTATAACAGTGATACTCTCGAGAGTATCACTGTTATAAGGTGCTTTTT and 2# CCGGCCAACAATTAGAAGCACCTTACTCGAGTAAGGTGCTTCTAATTGTTGGTTTTTG. The small interfering RNA (siRNA) duplexes that targeted p53 were 5′-CTACATGTGTAACAGTTCCUU-3′ and 5′-GGAACTGTTACACATGTAGUU-3′. An RNAi negative control (NC) was also used. Transfection was performed using Lipofectamine 3000 (Life Technologies), following the manufacturer’s protocol.

### Western blotting

All cells were lysed in 1× sodium dodecyl sulfate (SDS) loading buffer. Lysates containing equal amounts of protein were loaded onto each lane, followed by separation using 10% SDS polyacrylamide gel electrophoresis. Proteins were then transferred to 0.45-µm polyvinylidene fluoride membranes. The membranes were blocked at room temperature for 1 h in 5% FBS solution and subsequently incubated with primary antibodies at 4 °C overnight. The membranes were then incubated with secondary antibody for 1 h at room temperature and visualized using electrochemiluminescence. GAPDH was used as the loading control to normalize the intensity of the target protein bands. CD47 antibody (ab175388), PD-L1 antibody (ab213524), wild-type p53 antibody (ab26), mutant p53 antibody (ab32049), p21 antibody (ab109520), GAPDH antibody (ab9485), and goat anti-rabbit IgG H&L (ab6721) were purchased from Abcam.

### Flow cytometric assay

Single-cell suspensions were incubated with IgG antibody (17-4714-42, eBioscience) or CD47 (17-0479-42, eBioscience)/PD-L1(17-5983-42, eBioscience) antibody at 4 °C for 30 min in the dark. The cells were then washed using phosphate-buffered saline (PBS) and centrifuged at 1000 rpm for 5 min. Finally, the cells were resuspended and protein expression was analyzed on a FACSCalibur flow cytometer.

### Immunohistochemistry (IHC)

Formalin-fixed, paraffin-embedded cervical specimens representing a wide range of cervical disease processes, including 15 normal cervical tissue, 85 cervical intraepithelial neoplasia (CIN) tissues, and 101 cervical cancer tissues, were collected from the case files of Shanghai Jiao Tong University Affiliated Shanghai General Hospital between 2008 and 2018. The collection of human specimens and all experimental protocols were approved by the Ethics Committee of the School of Medicine of Shanghai Jiao Tong University, and written informed consent was obtained from all subjects in accordance with the Declaration of Helsinki. Each section was evaluated based on the percentage of positively stained cells (0–4) and the intensity of the staining (0–3). Finally, these two scores were multiplied together, giving a total score of up to 12 points. The higher the score, the more protein the sample expressed.

### Chromatin immunoprecipitation quantitative real-time PCR (ChIP-qPCR)

ChIP assays were performed as previously^2^ described. Briefly, cells were collected after cross-linking with 1% formaldehyde and then disrupted with ultrasound and incubated with H3K4me2 antibodies overnight. The immunoprecipitates were washed continuously to remove non-specific binding. After reverse cross-linking, the DNA samples were purified and analyzed by Quantitative real-time PCR (qRT-PCR). The specific primer sequences for the CD47/CD274 promoters are given in Supplementary Data 1.

### Quantitative real-time PCR

Total RNA from cell lines was extracted using TRIzol reagent (TaKaRa, Dalian, China). Complementary DNA was synthesized with random primers or microRNA-specific stem-loop primers (RiboBio) using a PrimeScript RT Reagent Kit (TaKaRa). Subsequently, qRT-PCR was performed with SYBR Premix Ex Taq (TaKaRa) on a 7500 real-time PCR system (Applied Biosystems, Mannheim, Germany). The 2^−ΔΔCt^ method was used to calculate relative expression levels using GAPDH or U6 as the endogenous control. The mRNA primer sequences are listed in Supplementary Data 2.

### Luciferase reporter assay

The 3′ untranslated region (3′UTR) of CD47/PD-L1 containing the wild-type or mutated binding sites of miR-34a were generated by Genomeditech (Shanghai, China). Dual-reporter luciferase assays were performed in the SiHa and HEK-293T cell lines. CD47/PD-L1 reporter constructs and miR-34a mimic or miR-NC were transfected into cells using Lipofectamine 3000, and luciferase activity was measured in cell lysates 24 h after transfection using a Luciferase Assay Kit (Promega, Madison, WI, USA).

### Animal study

Six-to-7-week-old female C57BL/6 mice (Animal Breeding Facility of the China Academy of Science, Shanghai, China) were housed and maintained in accordance with the standards of the National Institutes of Health guide for the care and use of laboratory animals (NIH Publications No. 8023, revised 1978). All experimental protocols were approved by the Ethics Committee of the School of Medicine of Shanghai Jiao Tong University. TC-1 cells were trypsinized and suspended in PBS, then 5 × 10^6^ cells were injected subcutaneously into mice to investigate tumorigenicity. Mice were randomly divided into four groups with four mice for each group. ORY-1001 (50 mg/kg) and anti-CD47 (100 µg/mouse)/PD-L1 (300 µg/mouse) monoclonal antibodies (mAbs) were administered intraperitoneally three times per week from the fifth day after tumor implantation. Three to 4 weeks after implantation, mice were sacrificed by cervical dislocation after inhalation of isoflurane, and the tumors were removed, dissected, and weighed blindly.

### Statistical analysis

Values are expressed as mean ± SD. Analysis of variance and two-tailed Student’s *t* tests were used to calculate *p* values. A *p* value < 0.05 was considered statistically significant.

## Results

### LSD1 expression is positively correlated with CD47/PD-L1 expression in cervical cancer cells and tissues

**Fig. 1:**
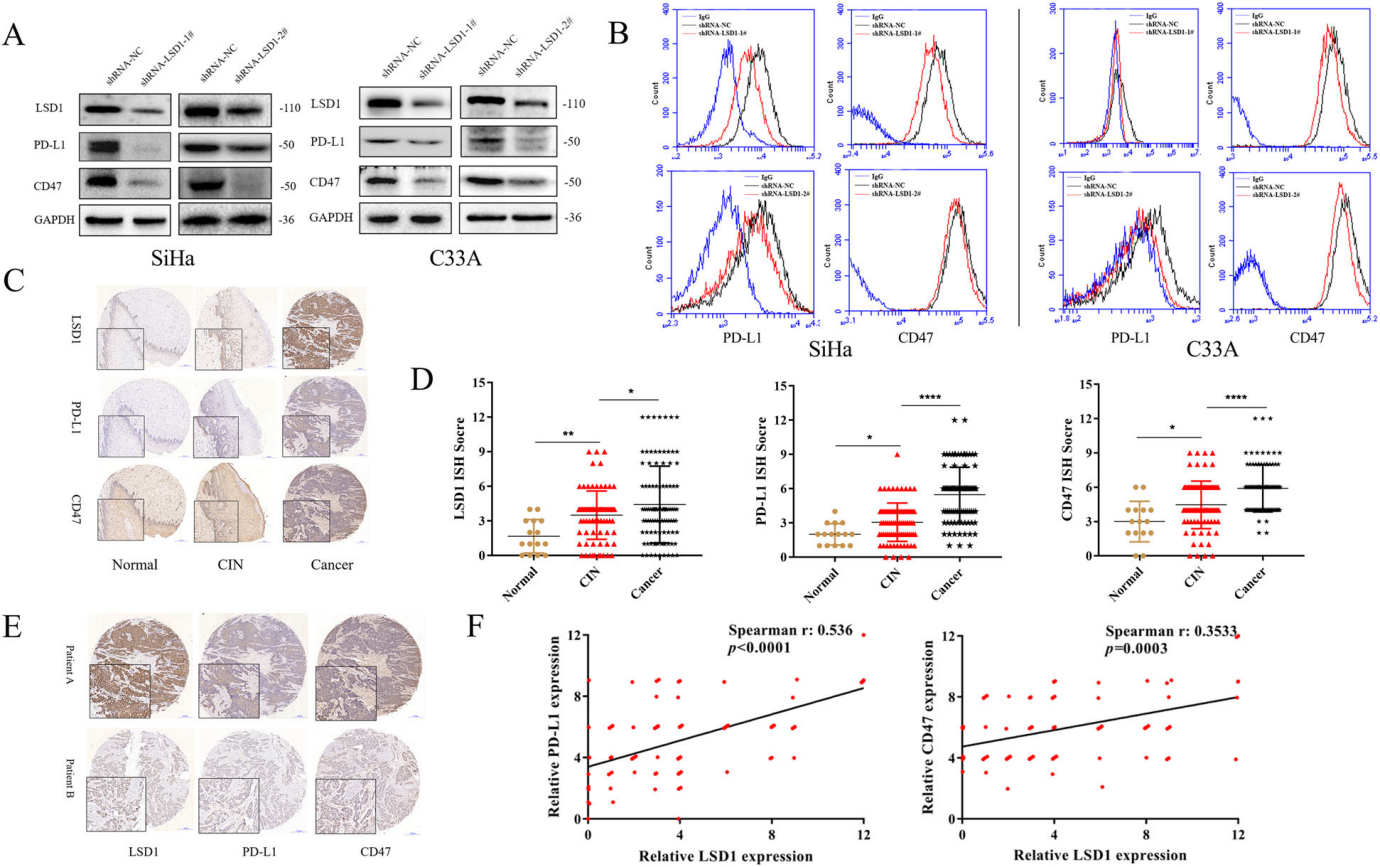
LSD1 is positively correlated with CD47/PD-L1 in cervical cancer cells and tissues. The positive correlation between LSD1 and CD47/PD-L1 expression was demonstrated using western blotting (**A**) and flow cytometry (**B**). Both IHC staining intensity (**C**) and IHC score (**D**) for LSD1, as for CD47/PD-L1, showed a steady increase during the progression of cervical cancer. IHC staining intensity (**E**) and Spearman correlation analysis (**F**) also confirmed the significant correlation between LSD1 and CD47/PD-L1 expressionfrom a clinical perspective. The western blotting and flow cytometric processes were repeated three times. *p < 0.05; **p < 0.01; ****p < 0.0001.

After the LSD1 silencing plasmid was transfected into SiHa and C33A cells, significant downregulation of CD47/PD-L1 protein expression was observed by western blotting (Fig. 1A) and flow cytometry (Fig. 1B). To further confirm the correlation between LSD1 expression and CD47/PD-L1 expression, we performed 201 instances of immunohistochemistry staining in tissue microarrays, including normal cervical tissues, CIN tissues, and cervical cancer tissues. The protein expression (Fig. 1C) and positive ratio (Supplementary Data 3) of LSD1 increased steadily, as did those of the CD47 and PD-L1 proteins, in the progression from normal cervical to CIN and then to cervical cancer. In addition, it was apparent that cervical cancer tissues with high LSD1 expression also had high CD47/PD-L1 expression (Fig. 1D). Consistently, Spearman correlation analysis showed that the expression of LSD1 was positively correlated with that of CD47 and PD-L1 (Fig. 1E). Thus, the IHC results further validate our research from a clinical perspective.

### LSD1-mediated H3K4 demethylation in the *CD47*/*CD274* promoter region

**Fig. 2:**
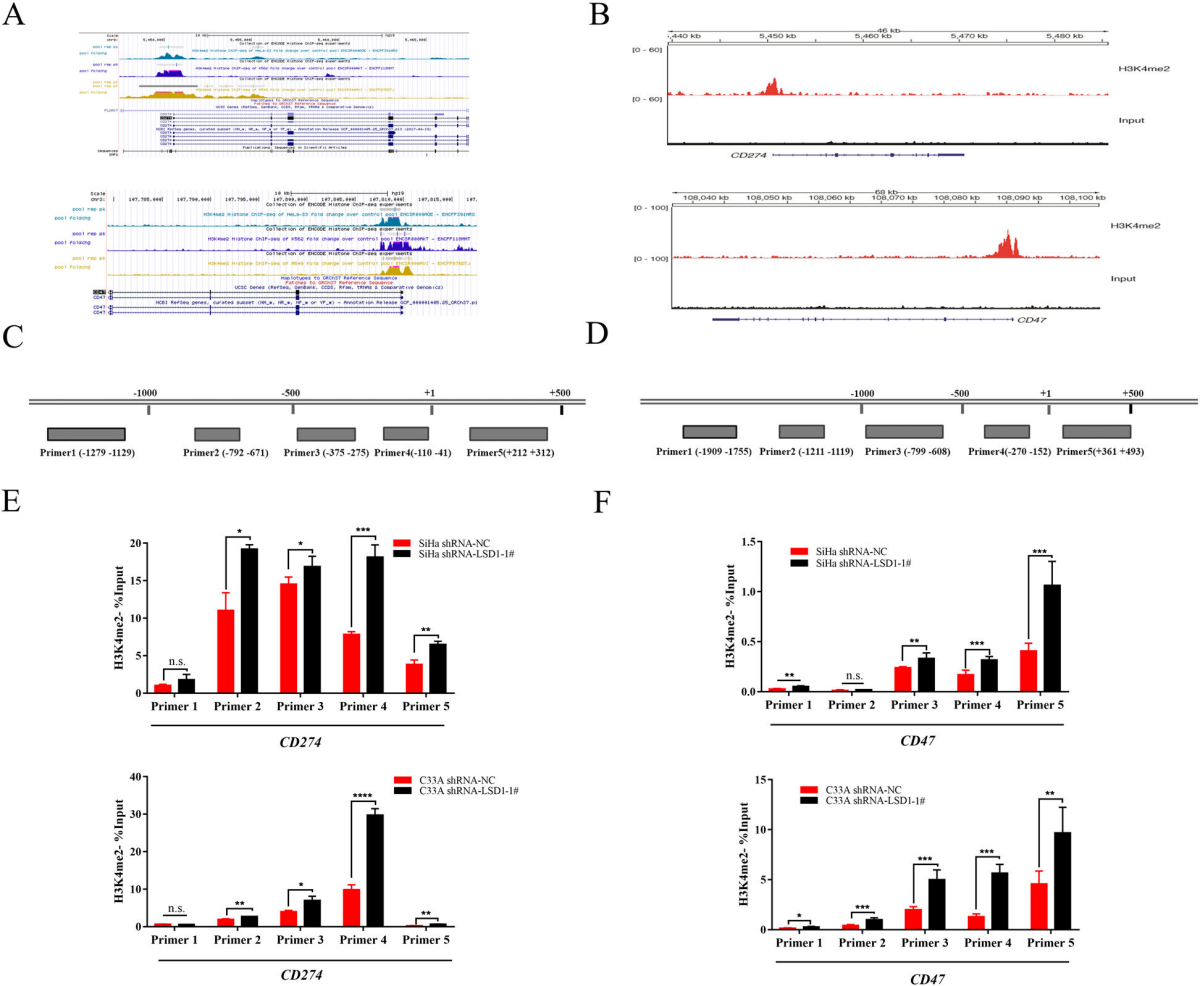
LSD1-mediated H3K4 demethylation in the *CD274*/*CD47* promoter region. **A** High abundance of H3K4me2 aggregation occurred in the *CD274*/*CD47* promoter region in a variety of tumor cells, including cervical cancer, according to histone ChIP-Seq data from the ENCODE database. **B** ChIP-Seq performed for SiHa cells also confirmed universal enrichment of H3K4me2 marks in the *CD274*/*CD47* promoter region. **C**, D Specific sequences targeting the *CD274*/*CD47* promoter region. **E** ChIP-qPCR indicated that the H3K4me2 level in the *CD274* promoter region increased significantly after LSD1 knockdown in SiHa and C33A cells (*n* = 3 per group); **F** the H3K4me2 level in the *CD47* promoter region underwent the same change (*n* = 3 per group). ChIP-qPCR experiments were repeated twice. N.S. not significant. *p < 0.05; **p < 0.01; ***p < 0.001; ****p < 0.0001.

To further access the epigenetic mechanisms responsible for the downregulation of CD47 and PD-L1 expression after LSD1 knockdown in cervical cancer cells, we obtained visualization data of histone ChIP sequencing (ChIP-Seq) from the ENCODE database (https://www.encodeproject.org/) and observed enrichment of H3K4me2 marks in the *CD47/CD274* promoter region in various tumor cells (Fig. 2A). In the subsequent ChIP-Seq, enriched H3K4me2 marks were also detected in the *CD47*/*CD274* promoter region in SiHa cells, consistent with the ENCODE data (Fig. 2B). For further confirmation, SiHa and C33A cells were then transfected with scrambled and LSD1-specific shRNA-coding lentivirus, and stable cells were selected. In addition, five specific ChIP-qPCR primers targeting the *CD47*/*CD274* promoter region were designed (Fig. 2C, D). ChIP analysis subsequently showed that silencing LSD1 expression markedly increased H3K4me2 levels in the *CD47*/*CD274* promoter regions compared with no-load stable SiHa and C33A cells (Fig. 2E, F). This suggested that CD47 and PD-L1 expression could be directly regulated through LSD1-mediated H3K4 demethylation in cervical cancer.

### LSD1 silencing reduces CD47/PD-L1 protein expression by upregulation of miR-34a

**Fig. 3:**
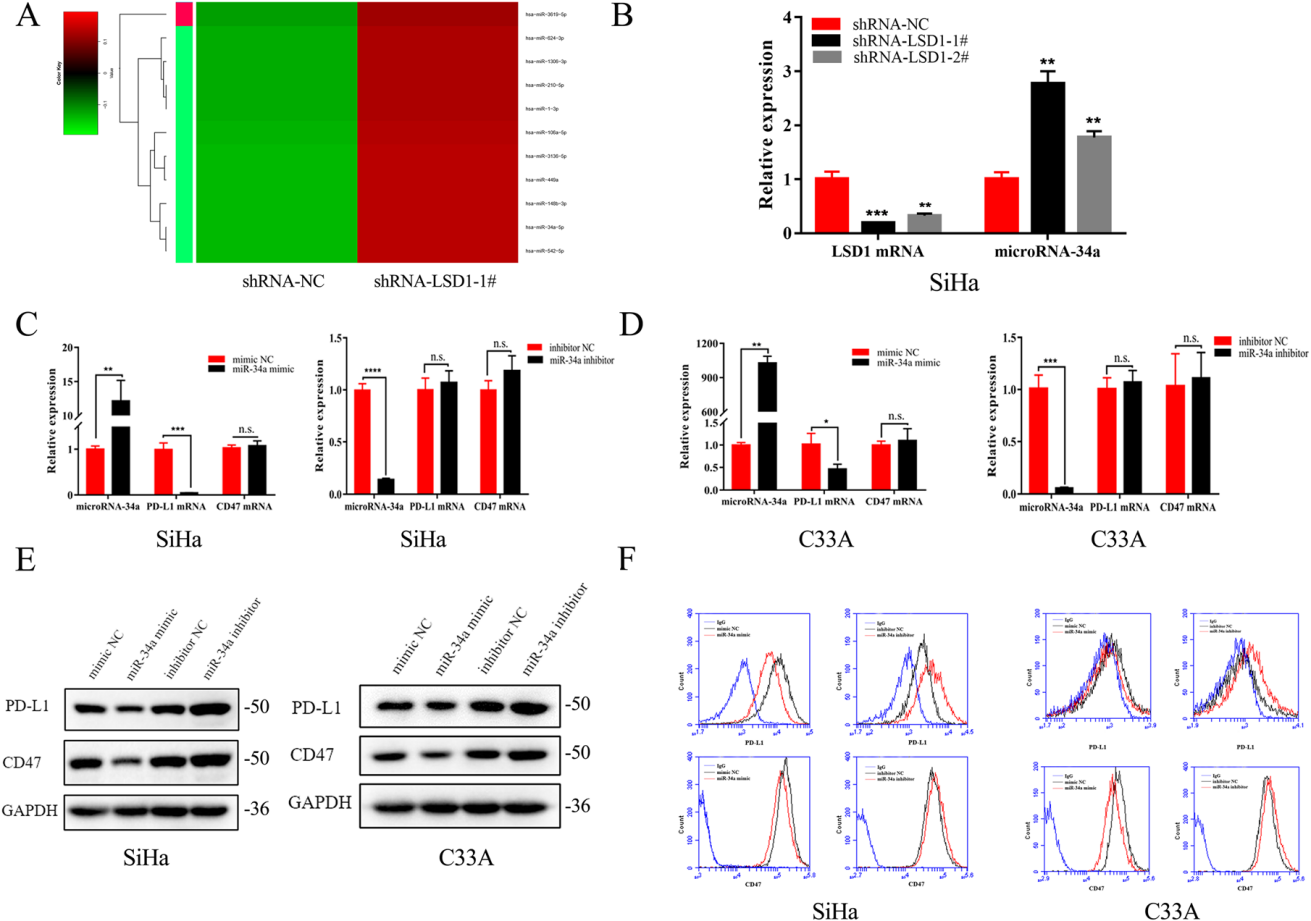
LSD1 silencing reduces CD47/PD-L1 protein expression by upregulation of miR-34a. **A** MicroRNA sequencing indicated that miR-34a, which was predicted to target the 3′UTR of CD47/PD-L1, was upregulated significantly after LSD1 knockdown in SiHa cells. **B** Changes in miR-34a expression after LSD1 knockdown in SiHa cells were confirmed by qRT-PCR (*n* = 3 per group). **C**, **D** Changes in the expression of PD-L1/CD47 mRNA in SiHa and C33A cells after transfection with miR-34a mimics or inhibitors (*n* = 3 per group). **E** Western blotting and **F** flow cytometry showed that miR-34a mimics upregulated the protein expression of CD47/PD-L1 in SiHa and C33A cells, whereas miR-34a inhibitors had the opposite effects. Each experiment was repeated three times. N.S. not significant. *p < 0.05; **p < 0.01; ***p < 0.001; ****p < 0.0001.

We next asked whether certain upregulated microRNAs might target CD47/PD-L1, resulting in translational suppression of CD47/PD-L1 mRNA in cells transfected with LSD1-shRNA. Therefore, we conducted microRNA sequencing and analyzed differentially regulated microRNAs of SiHa cells transfected with shRNA-NC or shRNA-LSD1 (Fig. 3A). When LSD1 was downregulated, miR-34a expression was strongly upregulated; this was further confirmed by qRT-PCR in SiHa cells (Fig. 3B). However, no significant change in the expression of miR-34a was observed in C33A cells after LSD1 knockdown (Supplementary Fig. 1A).

**Fig. 4:**
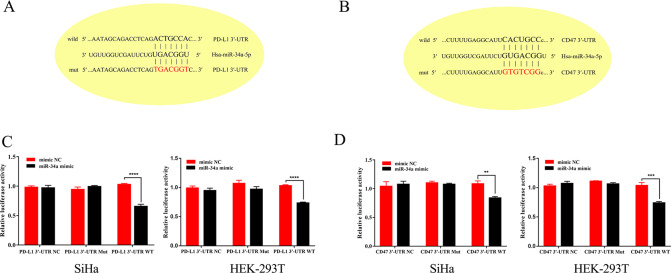
miR-34a inhibits the translation of CD47/PD-L1 mRNA through targeting the 3′UTR of the CD47/PD-L1. **A**, **B** The seed region of miR-34a has a highly complementary sequence to the 3′UTR of CD47/PD-L1. **C**, **D** Luciferase reporter assays were performed in HEK-293T and SiHa cells with co-transfection of the negative control, wild-type, or mutant 3′UTR constructs and mimic NC or miR-34a mimic (*n* = 3 per group). Each experiment was repeated twice. N.S. not significant, **p < 0.01; ***p < 0.001; ****p < 0.0001.

In order to verify the regulation of CD47/PD-L1 by miR-34a, miR-34a mimics and inhibitors were transfected into both SiHa and C33A cells. Consistent with our prediction, PD-L1 mRNA was significantly reduced after transfection of miR-34a mimics, whereas no significant change was observed after inhibition of miR-34a (Fig. 3C, D). There was no significant change in CD47 mRNA levels after transfection with either miR-34a mimics or inhibitors (Fig. 3C, D). However, the protein expression of CD47/PD-L1 was decreased when miR-34a was overexpressed in SiHa and C33A cells, and an increase in CD47/PD-L1 protein was observed due to the inhibition of miR-34a (Fig. 3E, F). We predicted that CD47/PD-L1 is a potential target of miR-34a, given the considerable complementarity between the seed region of miR-34a and the 3′UTR of CD47/PD-L1 (Fig. 4A, B). Finally, dual-reporter luciferase assays were carried out in SiHa and HEK-293T cells to determine whether miR-34a could bind to the 3′UTRs of CD47 and PD-L1. Transfection with the miR-34a mimic reduced the luciferase activity of the construct compared with mimic NC-transfected cells. Meanwhile, miR-34a showed no effect on the luciferase activity of site-directed mutagenesis of the putative miR-34a-binding sites in these constructs (Fig. 4C, D).

### LSD1 silencing increased miR-34a expression through targeting wild-type p53

We next asked how LSD1 plays its part in the regulation of miR-34a. It is generally known that the miR-34 family is a direct transcriptional target of wild-type p53, and miR-34 dysregulation has been hypothesized to be involved in the development of some cancers containing wild-type p53^12,13^. Wild-type p53 is present in a stable complex with LSD1 according to previous observations^14^. In addition, it has been shown that the activity of wild-type p53 and the transcription it targets are repressed by LSD1 through specifically demethylating both K370me1 and K370me2 in vitro^14^. Therefore, it is reasonable to speculate that wild-type p53 may be the key component involved in LSD1-mediated miR-34a expression in cervical cancer cells.

**Fig. 5:**
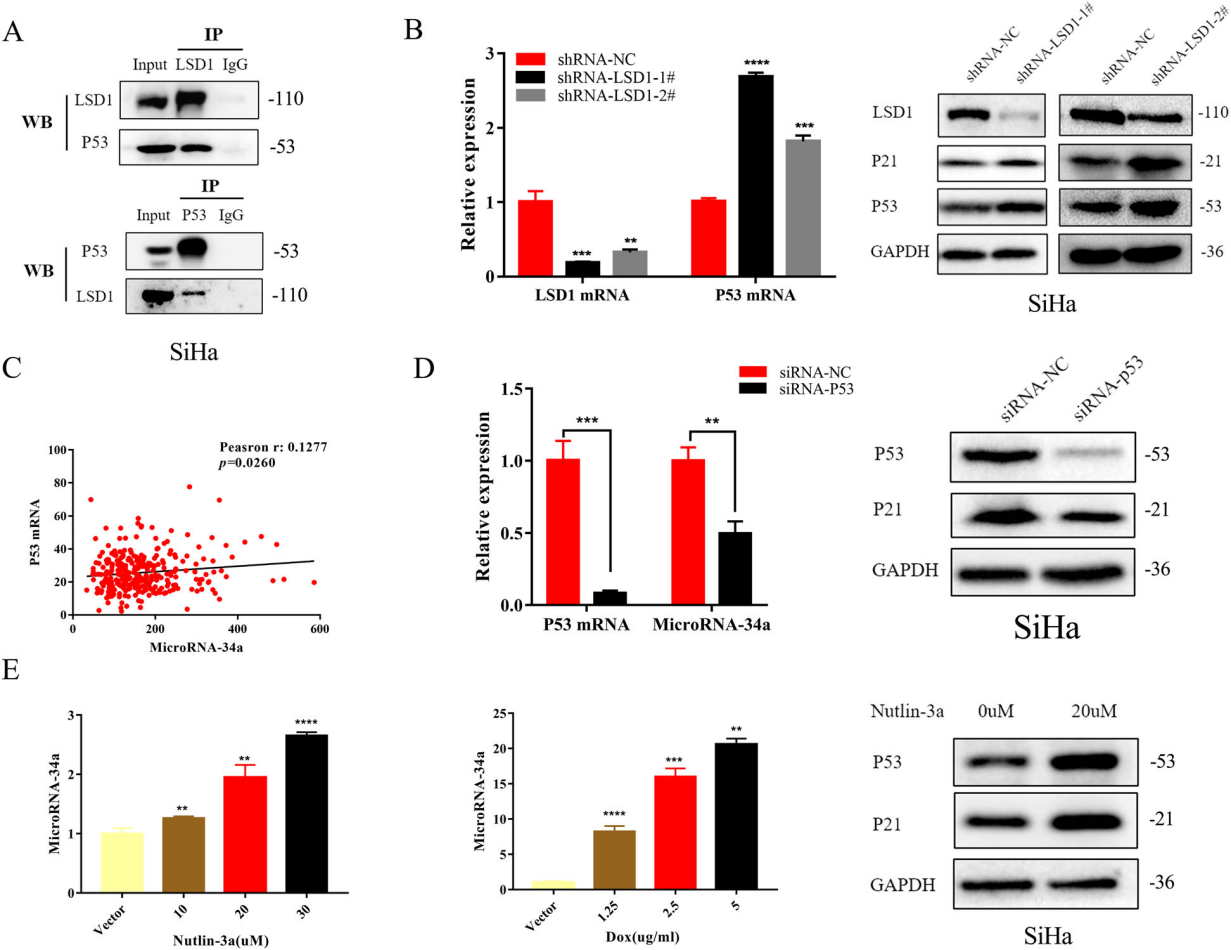
LSD1 silencing increased miR-34a expression through targeting wild-type p53. **A** Co-IP assay showed that LSD1 formed a stable complex with wild-type p53 in SiHa cells. **B** LSD1 knockdown contributes to upregulation of p53 mRNA and protein expression in SiHa cells (*n* = 3 per group). **C** There was a positive correlation between miR-34a and p53 mRNA in cervical cancer according to the normalized counts RNA-sequencing data from TCGA-CESC. **D** p53 silencing led to downregulation of miR-34a expression in SiHa cells (*n* = 3 per group). **E** Nutlin-3a and doxorubicin were used to stimulate p53 in SiHa cells and contributed to upregulation of miR-34a expression in a dose-dependent manner (*n* = 3 per group). Each experiment was repeated three times. **p < 0.01; ***p < 0.001; ****p < 0.0001.

We further verified that LSD1 formed a stable complex with wild-type p53 in SiHa cells (Fig. 5A). In addition, increases in both mRNA and protein expression of wild-type p53 in SiHa cells were observed after LSD1 knockdown (Fig. 5B). Meanwhile, there was no significant change in either the expression of mutant p53 mRNA or protein in C33A cells after LSD1 knockdown (Supplementary Fig. 1B). We further quantified the strength of the correlation between p53 and miR-34a expression using The Cancer Genome Atlas cervical squamous cell carcinoma and endocervical adenocarcinoma (TCGA-CESC) database. As shown in Fig. 5C, a positive correlation of p53 mRNA with miR-34a was found in the TCGA-CESC database. In addition, decreased miR-34a expression was observed by silencing wild-type p53 using siRNA-p53 in SiHa cells (Fig. 5D), and miR-34a expression increased in a dose-dependent manner when p53 was stimulated in SiHa cells using nutlin-3a and doxorubicin (Fig. 5E). Therefore, we speculated that LSD1 silencing increased miR-34a expression through targeting wild-type p53.

### LSD1 inhibitor enhances the efficacy of anti-CD47/PD-L1 immunotherapy therapy in a cervical cancer allograft model in vivo

**Fig. 6:**
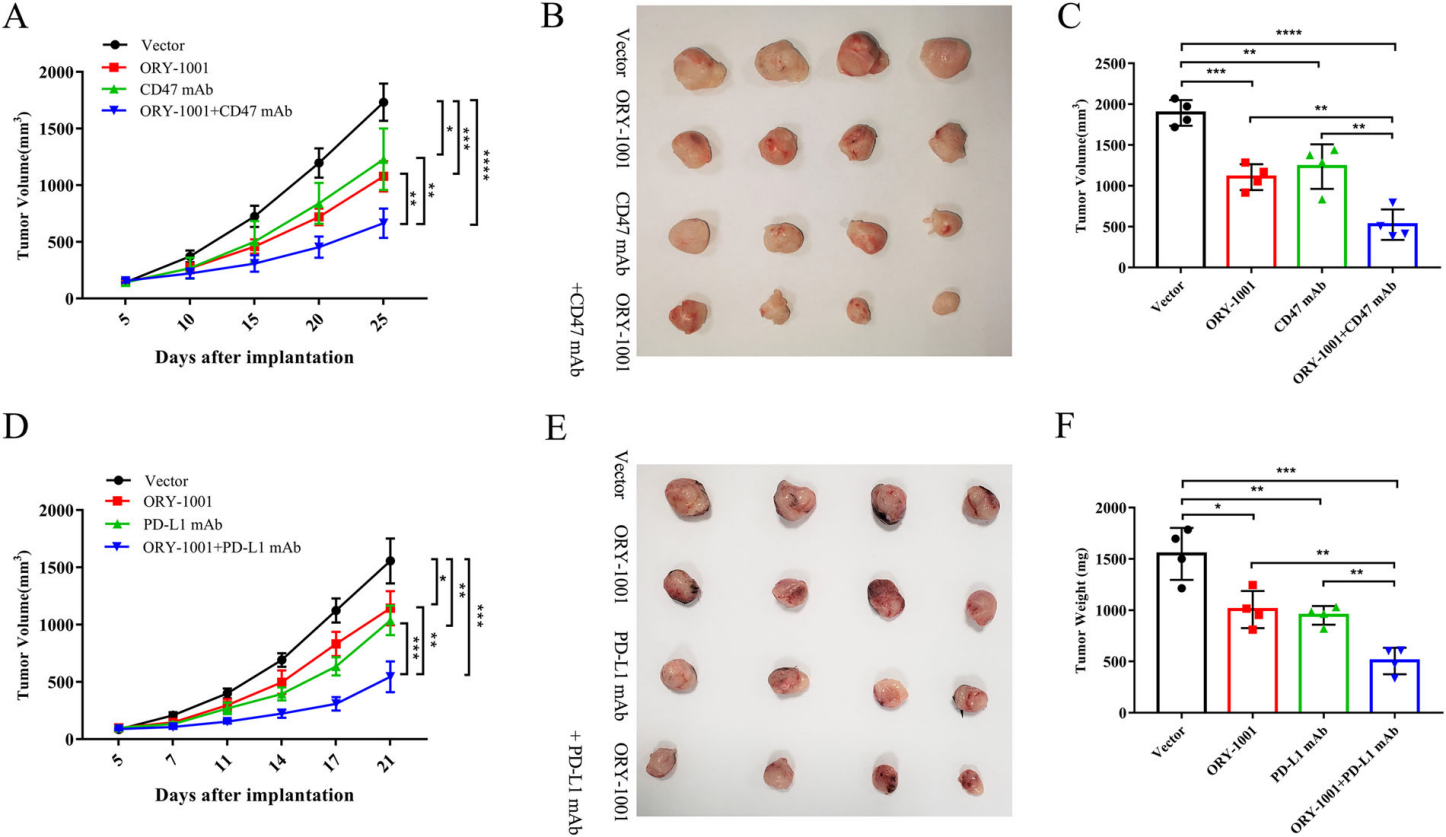
LSD1 inhibitor enhances the efficacy of anti-CD47/PD-L1 immunotherapy therapy in cervical cancer allograft model in vivo. **A** A combination of LSD1 inhibitors (50 mg/kg, three times a week) and anti-CD47 mAb (100 µg per mouse, three times a week) showed more significant inhibition of tumor growth than single drug administration (*n* = 4 per group). Evaluation of the appearance (**B**) and weights (**C**) of tumors in the four groups demonstrated the enhanced efficacy of anti-CD47 immunotherapy therapy when combined with an LSD1 inhibitor in vivo. **D**–**F** Enhanced treatment efficacy was also achieved with a combined administration of LSD1 inhibitors (50 mg/kg, three times a week) and anti-PD-L1 mAb (300 µg per mouse, three times a week) (*n* = 4 per group). *p < 0.05, **p < 0.01, ***p < 0.001, ****p < 0.0001.

To evaluate whether a combination of LSD1 inhibitors and anti-mouse CD47 mAb could reduce tumor burden in C57BL/6 mice subcutaneously inoculated with the TC-1 cells, 5 days after implantation, LSD1 inhibitors along with anti-mouse CD47 mAb were administered intraperitoneally three times per week. Compared with the NC animals, administration of an LSD1 inhibitor or anti-mouse CD47 mAb alone inhibited the growth of tumors. The combined use of the LSD1 inhibitor and anti-mouse CD47 mAb significantly enhanced the inhibition of tumor growth (Fig. 6A). Differences in appearance (Fig. 6B) and weight (Fig. 6C) of tumors among the four groups were also observed. Similar results were obtained with a combination of LSD1 inhibitor and anti-mouse PD-L1 mAb (Fig. 6D–F).

## Discussion

LSD1 is highly expressed in various tumors and is closely related to tumor progression. It is capable of specifically removing methyl groups from monomethylated and demethylated histone H3 lysine 4 (H3K4me1/2) and lysine 9 (H3K9me1/2). It is well recognized that the innate immune regulator CD47 and the adaptive immune checkpoint PD-L1 regulate tumor immune responses through the SIRPα/CD47 and PD-1/PD-L1 pathways, respectively. Tumor immunotherapies based on CD47/PD-L1 blockade have shown strong antitumor effects and have been demonstrated to prolong survival when used in the treatment of various tumors.

Our study demonstrated that LSD1 knockdown could lead to decreased protein expression of CD47/PD-L1. Meanwhile, the expression of LSD1 in cervical cancer tissues showed a highly positive correlation with CD47/PD-L1. These results preliminarily demonstrated the regulation of CD47/PD-L1 expression by LSD1.

Afterwards, we conducted in-depth verification of the mechanism by which LSD1 regulates CD47/PD-L1 expression. ChIP-Seq traces generated from the ENCODE database showed a large accumulation of H3K4me2 in the *CD47*/*CD274* promoter regions of many tumor cells. Similar results were observed in SiHa cells in subsequent ChIP-Seq experiments. Moreover, H3K4me2 levels in the *CD47*/*CD274* promoters of SiHa and C33A cells further increased after LSD1 knockdown, suggesting a direct regulatory effect of LSD1-mediated H3K4 demethylation on CD47 and PD-L1 expression in cervical cancer.

In addition, miR-34a, which is predicted to target the 3′UTR of CD47/PD-L1, increased significantly after LSD1 knockdown in SiHa cells. Thereafter, the regulation of CD47/PD-L1 protein expression by miR-34a was confirmed by transfecting miR-34a mimics or inhibitors into cervical cancer cells. Finally, dual-reporter luciferase assays in SiHa and HEK-293T cells confirmed that miR-34a could bind to the 3′UTR of CD47/PD-L1 and suppress the expression of CD47/PD-L1.

It was further verified that LSD1 formed a stable complex with wild-type p53 in SiHa cells and increases in both mRNA and protein expression of wild-type p53 in SiHa cells were observed after LSD1 knockdown. A positive correlation of p53 mRNA with miR-34a was demonstrated by silencing and stimulating wild-type p53 in SiHa cells. Therefore, we identified a crucial role of wild-type p53 in LSD1-mediated miR-34a expression in cervical cancer cells.

Based on these results, we treated C57BL/6 mice bearing TC-1 tumors to explore whether LSD1 inhibitors could enhance the efficacy of CD47/PD-L1 blockade therapy in vivo. The results showed that LSD1 inhibitors combined with anti-mouse CD47/PD-L1 mAb significantly inhibited tumor growth and altered the tumor microenvironment, indicating that LSD1 may contribute to tumor immune escape through the suppression of immune surveillance against tumor cells. After LSD1 knockdown, the loss of “do not eat me” and “do not find me” signals promotes immune activation and thus leads to inhibition of tumor growth.

In conclusion, our study revealed that LSD1 contributes to cervical cancer immune escape through regulation of CD47/PD-L1 protein expression. In addition, the LSD1-H3K4me2-CD47/PD-L1 and LSD1-wtp53-miR-34a-CD47/PD-L1 signaling axes were demonstrated to explain the direct and indirect regulation of CD47/PD-L1 by LSD1. Moreover, inhibition of LSD1 in combination with anti-mouse CD47/PD-L1 mAb could significantly suppress the growth of an allograft model by enhancing the immunogenicity of cervical cancer in vivo. In short, these results indicate that LSD1 is a potential target for enhancing the efficacy of immunotherapy for cervical cancer in the future.

## Supplementary information

Supplementary Figure 1

Figure legends for Supplementary Fig. 1.

Primer sequences targeting the CD47/CD274 promoter region

The mRNA primer sequences used in the study

The relative expression of LSD1, CD47 and PD-L1 protein in the issues of normal cervix, CIN and cervical cancer

## Data Availability

The datasets used and/or analyzed during the current study are available from the corresponding author on reasonable request.
