# Supplementary figures and images for: LSD1 silencing contributes to enhanced efficacy of anti-CD47/PD-L1 immunotherapy in cervical cancer
Source: Cell Death Dis. 2021 Mar 17;12(4):282. doi: 10.1038/s41419-021-03556-4 (PMC7969769; doi:10.1038/s41419-021-03556-4)

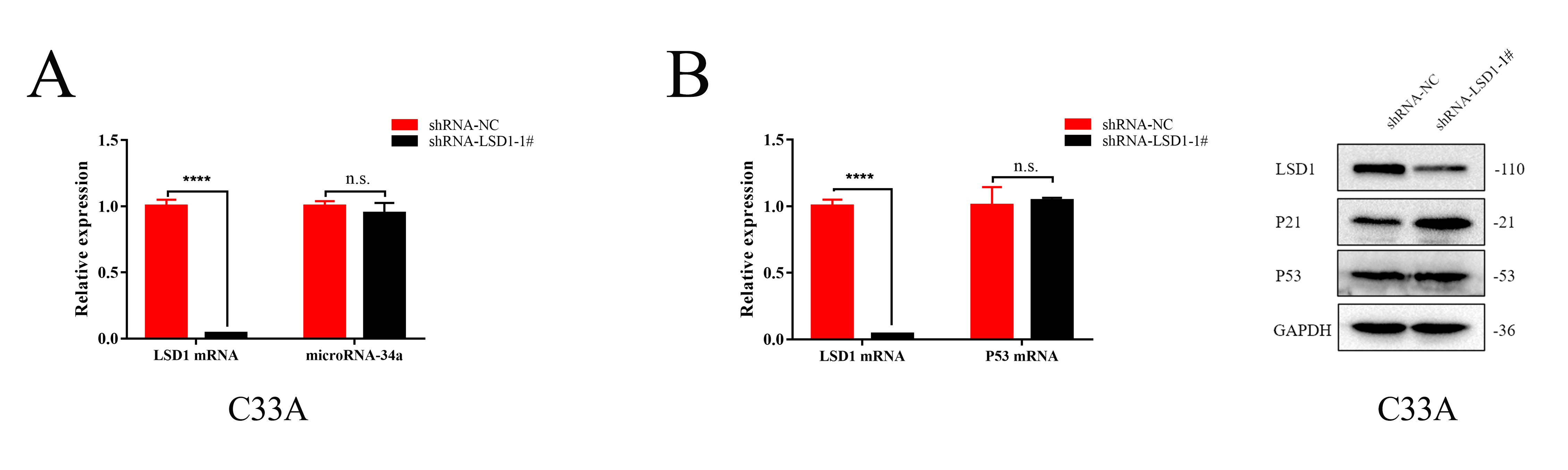

Supplement: Supplementary file 1 — Supplementary Figure 1 [file 41419_2021_3556_MOESM1_ESM.tif]
