# Supplementary material for: LSD1 silencing contributes to enhanced efficacy of anti-CD47/PD-L1 immunotherapy in cervical cancer
Source: Cell Death Dis. 2021 Mar 17;12(4):282. doi: 10.1038/s41419-021-03556-4 (PMC7969769; doi:10.1038/s41419-021-03556-4)
Supplement: Supplementary file 2 — Figure legends for Supplementary Fig. 1. [file 41419_2021_3556_MOESM2_ESM.docx]

Supplementary Fig 1. (A) No significant change in the expression of miR-34a was observed in mutant p53-expressing C33A cells after LSD1 knockdown (n=3 per group). (B) Meanwhile, there was no significant change in either the expression of mutant p53 mRNA (n=3 per group) or protein in C33A cells after LSD1 knockdown. Each experiment was repeated three times. N.S., not significant. ****p < 0.0001.
