# Supplementary material for: LSD1 silencing contributes to enhanced efficacy of anti-CD47/PD-L1 immunotherapy in cervical cancer
Source: Cell Death Dis. 2021 Mar 17;12(4):282. doi: 10.1038/s41419-021-03556-4 (PMC7969769; doi:10.1038/s41419-021-03556-4)
Supplement: Supplementary file 3 — Primer sequences targeting the CD47/CD274 promoter region [file 41419_2021_3556_MOESM3_ESM.docx]

Supplementary data 1

Primer sequences targeting the *CD47*/*CD274* promoter region

| Primer name | Sequences (5′ to 3′) |
| --- | --- |
| *CD47*-1-forward | TTTTGCCCCACCACCAAAAC |
| *CD47*-1-reverse | AGGTGGAGATCGGGTGATGT |
| *CD47*-2-forward | GTGCCACTTTCATACGCTGG |
| *CD47*-2-reverse | TTCCCACCACAATACCCCAC |
| *CD47*-3-forward | TGGGGATGTGTTGGATACGC |
| *CD47*-3-reverse | CTCTGCTCTTCCCTATGCGG |
| *CD47*-4-forward | ACAGGAACGGGTGCAATGAG |
| *CD47*-4-reverse | CTTCCAGGTCACGTCCTGTC |
| *CD47*-5-forward | GAAGTGCAGACGTGGGAGG |
| *CD47*-5-reverse | TTTGGAGATGGAGAACCGGG |
| *CD274*-1-forward | AGAAGTTCAGCGCGGGATAA |
| *CD274*-1-reverse | GGCTGCGGAAGCCTATTCTA |
| *CD274*-2-forward | TTATCAGAAAGGGGGACGCC |
| *CD274*-2-reverse | ACAAGCCAACATCTGAACGC |
| *CD274*-3-forward | CCTAAACTGAAAGCTTCCGCC |
| *CD274*-3-reverse | GCAAATCCAGTTTGCCGGG |
| *CD274*-4-forward | CAACTTCGGGAACTTTGGGA |
| *CD274*-4-reverse | ATGGAGTTCTCTTTGGCCCC |
| *CD274*-5-forward | AAAGGGAACGCGATGGTCTA |
| *CD274*-5-reverse | AAACTGAATCGCGCCTGGA |
