# Supplementary material for: LSD1 silencing contributes to enhanced efficacy of anti-CD47/PD-L1 immunotherapy in cervical cancer
Source: Cell Death Dis. 2021 Mar 17;12(4):282. doi: 10.1038/s41419-021-03556-4 (PMC7969769; doi:10.1038/s41419-021-03556-4)
Supplement: Supplementary file 4 — The mRNA primer sequences used in the study [file 41419_2021_3556_MOESM4_ESM.docx]

Supplementary data 2

The mRNA primer sequences used in the study

| \| Primer name \| \| --- \| | \| Sequences (5′ to 3′) \| \| --- \| |
| --- | --- | --- | --- |
| GAPDH-forward | GGCACCGTCAAGGCTGAGAAC |
| GAPDH-reverse | GGTGGCAGTGATGGCATGGAC |
| LSD1-forward | AAGCAGGAGGACTTCAAGAC |
| LSD1-reverse | GCAGTGTGCGGTTTCTAATG |
| PD-L1-forward | GACCACCACCACCAATTCCAAGAG |
| PD-L1-reverse | TGAATGTCAGTGCTACACCAAGGC |
| CD47-forward | ACAAGTCCACTGTCCCCACT |
| CD47-reverse | ACTGTCCCCAGAACAGGAGT |
| P53-forward | ATGGAGGAGCCGCAGTCAGATCCTA |
| P53- reverse | TAGCTGCCCTGGTAGGTTTTCTGGG |
