# Supplementary material for: LSD1 silencing contributes to enhanced efficacy of anti-CD47/PD-L1 immunotherapy in cervical cancer
Source: Cell Death Dis. 2021 Mar 17;12(4):282. doi: 10.1038/s41419-021-03556-4 (PMC7969769; doi:10.1038/s41419-021-03556-4)
Supplement: Supplementary file 5 — The relative expression of LSD1, CD47 and PD-L1 protein in the issues of normal cervix, CIN and cervical cancer [file 41419_2021_3556_MOESM5_ESM.docx]

Supplementary data 3

The relative expression of LSD1, CD47 and PD-L1 protein in the issues of normal cervix, CIN and cervical cancer

| Variables | Total | Relative LSD1 expression | | Relative CD47 expression | | Relative PD-L1 expression | |
| --- | --- | --- | --- | --- | --- | --- | --- |
|  | (n=201)  No. | Negative(n=25 )  No.(%) | Positive(n=76)  No.(%) | Negative(n=41 )  No.(%) | Positive(n=160)  No.(%) | Negative(n=88 )  No.(%) | Positive(n=113)  No.(%) |
| Normal | 15 | 13 (86.7) | 2 (13.3) | 9 (60) | 6 (40) | 14 (93.3) | 1 (0.7) |
| CIN | 85 | 35 (41.2) | 50 (58.8) | 22 (25.9) | 63 (74.1) | 51 (60) | 34 (40) |
| Cervical cancer | 101 | 38 (37.6) | 63 (62.4) | 10 (9.9) | 91 (90.1) | 23 (22.8) | 78 (77.2) |
